# Candida pelliculosa endophthalmitis after cataract surgery: a case report

**DOI:** 10.1186/1756-0500-7-169

**Published:** 2014-03-21

**Authors:** Haluk Esgin, Erkan Bulut, Çaglar Örüm

**Affiliations:** 1 Department of Ophthalmology, Faculty of Medicine, Trakya University, Edirne, Turkey

## Abstract

**Background:**

Here we report the first case of postoperative endophthalmitis due to *Candida pelliculosa* after cataract surgery. We describe the clinical management of this type of candida infection in the eye.

**Case presentation:**

A 57-year-old Turk man was seen at our clinic at the end of the first postoperative month after cataract surgery. He presented with eye redness, pain and decreased visual acuity. His ophthalmologic examination revealed moderate tyndall and a mild flare in the anterior chamber. Hypopyon in the capsular bag posterior to the intraocular lens was seen in the second postoperative month. Despite topical and subconjunctival bacterial endophthalmitis treatment, there was no improvement in the clinical situation. *Candida pelliculosa* was isolated from a sample culture obtained from the anterior chamber. Oral fluconazole could not be administered because of increased liver enzyme levels and intravenous amphotericin B could not be administered because of an allergic reaction. Intraocular lens explantation, pars plana vitrectomy and anterior chamber lavage by rupturing the posterior wall of the microabscesses were performed. Intravitreal and intracameral amphotericin B injections were given four times in addition to surgical interventions. The patient has been followed for 2 years and his best-corrected visual acuity was 0.4 at the last visit.

**Conclusion:**

Nearly 1 month after cataract surgery, a patient presented with eye redness and blurred vision, with corneal endothelial deposits, hypopyon in the capsular bag and microabscesses on the incision sites and corneal endothelium. *Candida pelliculosa* should be considered in patients showing these symptoms. Multiple intraocular amphotericin B (5 μg) administrations can be used safely even in cases with high sensitivity to systemic use. Rupturing the posterior wall of the abscesses on the corneal endothelium surgically with intraocular lens explantation and pars plana vitrectomy are recommended.

## Background

Endophthalmitis is a serious intraocular inflammatory disease and the most common form is postoperative endophthalmitis. The incidence of post-surgical endophthalmitis is nearly 0.093% and the organisms responsible are generally bacteria. Three percent of all cases with endophthalmitis after cataract surgery are due to fungi [1,2]. Fungi have been isolated from 21.8% of all culture positive postoperative endophthalmitis cases, and mostly *Aspergillus* spp. and *Candida* spp. were determined to be the cause of fungal endophthalmitis [3,4]. This is the first reported case with endophthalmitis due to *Candida pelliculosa.*

## Case presentation

A 57-year-old Turk male was seen in our clinic with complaints of blurred vision in his left eye. His visual acuity was 2/20 and he had a nucleocortical and posterior subcapsular cataract. A history of systemic hypertension and past coronary by-pass surgery was present. Uncomplicated phacoemulsification surgery and foldable posterior chamber intraocular lens (IOL) implantation was performed. 1 mg/0.1 ml intracameral cefuroxime sodium (Cefurol® 1.5 g/15 ml flacon, I.E. Ulagay) was given for prophylaxis. The eye was closed with tobramycin ophthalmic ointment 3 mg/g and dexamethasone ophthalmic ointment 1 mg/g. Postoperative best-corrected visual acuity (BCVA) was 16/20 with minimal corneal edema after the first week.

The patient complained of pain and eye redness for 4 days on the 27th day after surgery. His BCVA was also decreased to 10/20. Ophthalmic examination revealed ++++ Tyndall, no hypopyon and ++ flare in the anterior chamber. Intraocular pressure was 14 mmHg OD; 17 mmHg OS. Fundus examination results were normal and the patient had no pain. Late onset toxic anterior segment syndrome (TASS) was suspected. Dexamethasone sodium phosphate eye drops 0.1% (Onadron® collyrium 1 ml, I.E. Ulagay) were given hourly, netilmicin sulfate eye drops 0.38% (Netira® collyrium, SIFI) were given 4 times a day and cyclopentolate HCl eye drops 1% (Sikloplejin® collyrium, Abdi Ibrahim) were given 3 times a day as a topical treatment.

**Figure 1:**
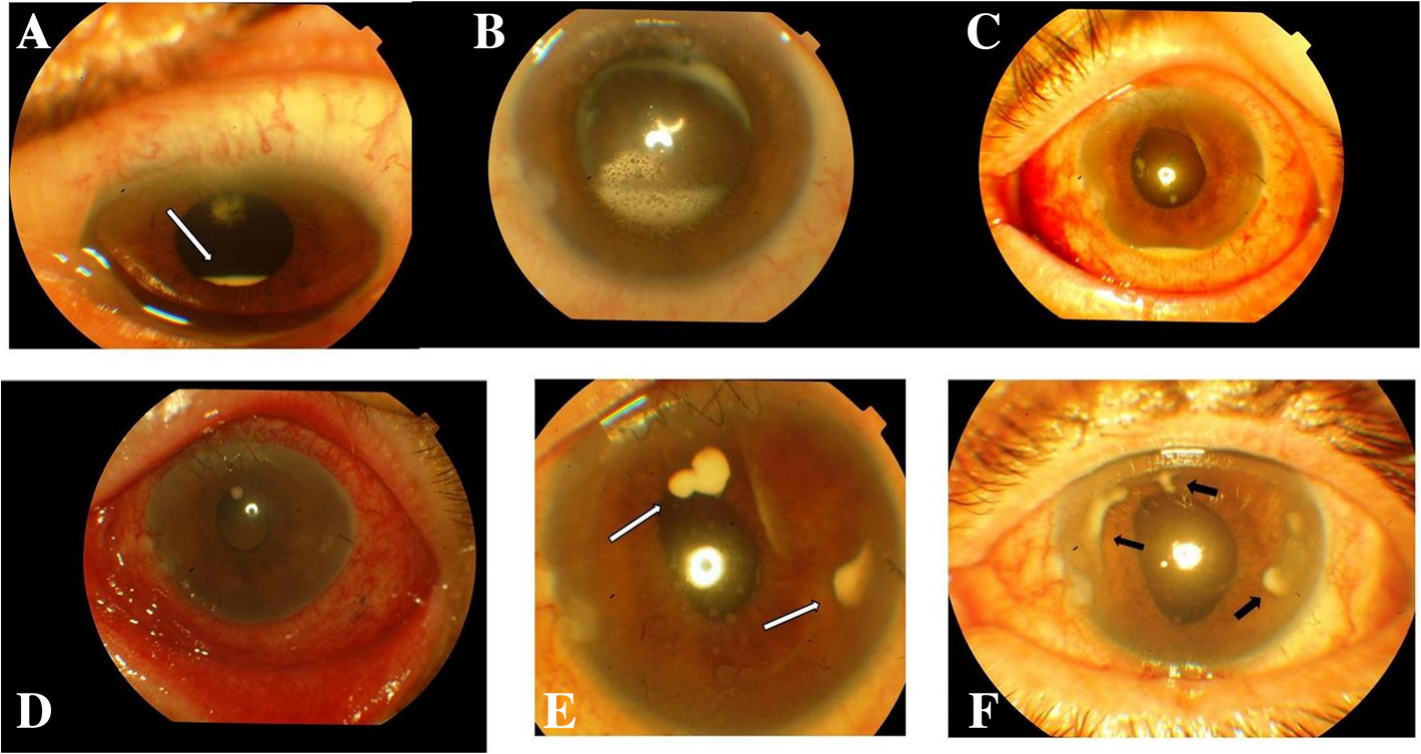
**Dense exudate (white arrow) in the inferior capsular bag. (A)**, endothelial precipitates and increased exudate in the capsular bag **(B)**, hypopyon and deposits on the anterior hyaloids **(C)**, corneal endothelial microabscesses **(D)**, increased corneal endothelial microabscesses (white arrows) **(E)**, sub endothelial encapsulated abscesses (black arrows) **(F)**.

Initially, the patient’s symptoms were under control, however, 3 weeks later, some deposits on the corneal endothelium and dense exudate in the inferior capsular bag were observed despite topical treatment (Figure 1A). Even after treatment, endothelial precipitates and exudates increased and the patient was hospitalized (Figure 1B). Visual acuity was not at the worst level (6/20) and the patient felt no pain. A fundus evaluation showed ++ tyndall and + flare. Because the endophthalmitis was not as severe, we decided to add subconjunctival vancomycin 25 mg/0.5 ml injection (Vancomycin® Hcl 1 g flacon, ORNA) twice a day. Ceftazidime 100 mg/0.5 ml injection (Fortum® 1 g flacon, GlaxoSmithKline) and dexamethasone 2 mg/0.5 ml injection (Dekort® 2 ml, 8 mg, Deva) were added to the topical treatment. gatifloxacin 0.3% eye drop hourly (Zymar® collyrium, Abdi Ibrahim), dexamethasone eye drop hourly (Maxidex® collyrium, Abdi Ibrahim) and cyclopentolate eye drop 3 times a day (Sikloplejin® collyrium, Abdi Ibrahim). On the 5th day of hospitalization, a sample was taken via paracentesis. Purulent material was aspirated from the capsular bag and anterior chamber while ceftazidime was injected. *C. pelliculosa* was isolated from the sample culture. Systemic antifungal therapy (fluconazole Tab 100 mg × 4 daily) was initiated but increased levels of liver enzymes (alanine aminotransferase: 461, aspartate aminotrasferase: 197) developed on the 4th day after starting fluconazole. The drug was stopped and intravenous infusion therapy with liposomal amphotericin B (L-amB; 5 mg/kg/day) was started. One minute following L-amB infusion, the patient began to feel pain and fever, with hyperemia in his neck region. The liposomal amB was then stopped because of this allergic reaction. The patient refused any other systemic therapy. We were reticent to initiate intravitreous administration because of the high sensitivity to antifungal agents and the side effects of intraocular amB. Then a topical treatment with fluconazole that was prepared using fluconazole flacon 100 mg/50 ml was started. Despite medical treatment, the inflammation persisted. An intraocular lens explantation was performed. Two weeks later, the BCVA decreased to 2/20 and the patient had 2 mm hypopyon and deposits on the anterior hyaloid with a blurred fundus (++++tyndall, +++flare; Figure 1C). A pars plana vitrectomy (PPV) and an intraocular amB injection (5 μg) were performed. Hypopyon was resolved and the deposits were decreased in number. Topical treatment with fluconazole, dexamethasone and cyclopentolate were continued. The BCVA increased to 8/20 and the fundus was blurry but normal.

One month after the PPV, the patient was observed to have ciliary flush, an increased anterior chamber reaction, corneal endothelial microabscesses and minimal optic nerve head swelling (Figure 1D). A second intravitreal amB ( 5 μg) dose was injected on that day. Even with topical fluconazole treatment, the patient had pain, decreased visual acuity and increased corneal endothelial microabscesses 1.5 months after the second intravitreal injection (Figure 1E). Therefore, anterior chamber lavage, aspiration of the abscesses on the endothelium and a third intracameral intravitreal amB (5 μg) dose with triamcinolone acetonide 4 mg were given. An increase in corneal endothelial microabscesses and pain occurred again after 1 month (Figure 1F). Then, anterior chamber lavage and aspiration of abscesses by rupturing the posterior wall were performed and intracameral amB was injected for the 4th time. In the fundus, there was a ++ vitreous cellular reaction and a pale optic nerve head. Thereafter, no relapse was observed. The Infection management took place for 8 months. Moreover, there was no recurrence of endophthalmitis during 3 years of follow-up. The BCVA of the patient remained at 8/20.

## Conclusions

Fungus is the cause of 7–13% of culture positive cases of postoperative endophthalmitis and the common fungal species that are isolated are *Aspergillus* spp., *Cephalosporium* spp. and *Candida* spp [2,3].

Possible sources of exogenous fungal infection are contaminated intraocular irrigation solutions [5], intraocular lenses [6], ventilation systems or hospital construction activities [7]. We could not find any predisposing factor to account for the fungal endophthalmitis in this case. Colonization at the time of cataract surgery or postoperative self-inoculation by the patient may have occurred.

The mean time interval between cataract surgery and the development of symptoms in exogenous fungal endophthalmitis is 19.5 days [3]. In candida endophthalmitis, it ranges from 3 days to 50 days [5,7]. The interval was 23 days in our *C. pelliculosa* case.

Corneal involvement, anterior chamber exudates, hypopyon and fibrinous reaction localized around the IOL have been seen in fungal endophthalmitis cases [7].

Parental antifungal treatment is recommended for 4–6 weeks in candida endophthalmitis. We were unable to manage allergic reactions due to L-AmB because of the hepatotoxicity of fluconazole and patient refusal. Increased liver enzymes due to fluconazole, such as in our case, have been seen in 2 of 27 cases [7].

Intraocular AmB injections might be needed several times in fungal endophthalmitis. In one case with exogenous fungal endophthalmitis, three intravitreal AmB injections were needed [8]. In another case with candida endophthalmitis, 67 mg amB was injected 11 times in total [9]. In a case series, two or more injections were performed in 2 of 27 eyes [7]. In our case, despite topical treatment, inflammation recurred and we performed intravitreal amB injections 4 times.

A combination of PPV and antifungal agents appears to be the best therapy for exogenous fungal endophthalmitis. The role of corticosteroids is controversial. Our aim in using corticosteroids was to diminish tissue destruction due to damage by fungal toxins.

It has been reported that visual outcomes were unfavorable in fungal postoperative endophthalmitis cases following PPV [3]. The final BCVA was better than 6/18 in only in 5 of 27 eyes [7]. Our final BCVA was 8/20 and we consider this a favorable outcome.

In summary, 23 days after cataract surgery, the patient presented with eye redness, pain and blurred vision. Corneal endothelial deposits, hypopyon in the capsular bag and microabscesses on the incision sites and corneal endothelium were thought to be caused by *C. pelliculosa*. PPV with IOL explantation and multiple amB (5 μg) injections into the anterior chamber and vitreous cavity with rupturing of the posterior wall of the abscesses on the endothelium are recommended.

## Consent

Written informed consent was obtained from the patient for publication of this Case Report and any accompanying images. A copy of the written consent is available for review by the Editor-in-Chief of this journal.

## Competing interests

The authors declare that they have no competing interests.

## Authors’ contributions

HE carried out the diagnosis, surgery and follow-up and suggested this case report, which he developed and coordinated. EB was the main physician responsible for caring for the patient and was involved in manuscript writing. ÇÖ performed retinal examinations and helped in reviewing the literature for this manuscript. All authors read and approved the final manuscript.
